# CD123 a Therapeutic Target for Acute Myeloid Leukemia and Blastic Plasmocytoid Dendritic Neoplasm

**DOI:** 10.3390/ijms24032718

**Published:** 2023-02-01

**Authors:** Elvira Pelosi, Germana Castelli, Ugo Testa

**Affiliations:** Department of Oncology, Istituto Superiore di Sanità, Viale Regina Elena 299, 00161 Rome, Italy

**Keywords:** acute myeloid leukemia, blastic plasmocytoid dendritic cell neoplasm, interleukin-3, interleukin-3 receptor, CD123, targeted therapy

## Abstract

In spite of consistent progress at the level of basic research and of clinical treatment, acute myeloid leukemia (AML) still represents an unmet clinical need for adult and pediatric patients. To improve the outcomes of these patients, it is necessary to identify new therapeutic targets. IL3RA (CD123, alpha subunit of the interleukin 3 receptor) is a cell membrane protein overexpressed in several hematologic malignancies, including AML blastic plasmocytoid dendritic cell neoplasms (BPDCN). Given the higher expression of CD123 on leukemic cells compared to normal hematopoietic cells and its low/absent expression on normal hematopoietic stem cells, it appears as a suitable and attractive target for therapy. Various drugs targeting CD123 have been developed and evaluated at clinical level: interleukin-3 conjugated with diphtheria toxin; naked neutralizing anti-CD123 antibodies; drug–antibody conjugates; bispecific antibodies targeting both CD123 and CD3; and chimeric antigen receptor (CAR) T cells engineered to target CD123. Some of these agents have shown promising results at the clinical level, including tagraxofusp (CD123 conjugated with diphtheria toxin) for the treatment of BPDCN and IMGN632 (anti-CD123 drug-conjugate), and flotetuzumab (bispecific anti-CD123 and anti-CD3 monoclonal antibody) for the treatment of AML. However, the therapeutic efficacy of CD123-targeting treatments is still unsatisfactory and must be improved through new therapeutic strategies and combined treatments with other antileukemic drugs.

## 1. Introduction

Acute myeloid leukemia (AML) is a heterogeneous malignant hematological disease characterized by the clonal proliferation of hematopoietic stem and progenitor cells (HSCPs) and blockade of differentiation of myeloid precursor cells that accumulate in bone marrow at the expense of normal hematopoiesis. The development of high-throughput sequencing techniques has consistently contributed to defining the genetic heterogeneity and complexity of AMLs, revising diagnostic and prognostic criteria, and identifying new therapeutic targets.

AMLs can be classified into three different groups depending on their origin: de novo, secondary (sAML), and therapy-related AMLs (tAML), which correspond to different clinical subtypes. According to the WHO classification of myeloid neoplasms: de novo AMLs are those occurring in the absence of prior predisposing events; sAMLs are defined as AMLs occurring after an antecedent myeloid neoplasia, such as myelodysplastic syndromes or myeloproliferative neoplasms; tAMLs are defined as AMLs occurring as the consequence of mutagenic events caused by cytotoxic chemotherapy and/or radiotherapy [1].

AMLs have been classified according to their clinico-biological properties [2]. The most adopted risk classification of AMLs is the ELN 2022 (European Leukemia Network) stratification that proposed the classification of AML patients into one of the three risk groups, such as favorable, intermediate, and adverse. The favorable prognosis group includes AMLs with acute promyelocytic leukemia (APL) t(15;17), balanced translocations t(8;21), biallelic mutated *CEBPA* and inv(16), and mutated *NPM1* without *FLT3-ITD*. The intermediate group comprises mutated *NPM1* with *FLT3-ITD*, WT-*NPM1* with *FLT3-ITD* (without adverse-risk genetic lesions), t(9;11), *MLLT3-MLL,* and cytogenetic abnormalities neither favorable nor adverse. The adverse AML group comprises AMLs with complex karyotype, inv(3)(q21q26)/t(3;3)(q21;q26), *DEK-NUP214* t(6;9)(p23;q34), *RPN1-EVI1*, *BCR-ABL1* t(9;22)(q34.1;q11.2), *KATA6-CREBBP* t(8;16)(p11.2;p13.3), t(6;11), −5 or del(5q), −7 or abnormal (17p) or monosomal karyotype, *TP53* mutations, *RUNX1*, *ASXL1, BCOR, EZH2, SF3B1, STAG2*, *U2AF1*, and *ZRS2* mutations [2].

Considerable progress has been made in the characterization of the molecular abnormalities underlying AMLs with the identification of recurrent chromosomal alterations and gene mutations, allowing the classification of these leukemias into various subgroups characterized by different genetic alterations and responses to current treatments [3,4,5,6,7].

The development of molecular analysis of AMLs has provided new fundamental knowledge on molecular pathogenesis of these disorders in genomic diagnostics and in the assessment of measurable residual disease; furthermore, these studies have greatly contributed to the identification of therapeutic targets and of new therapeutic agents, such as FLT3, IDH2, IDH2, and BCL2 inhibitors [8,9]. However, in spite of this consistent progress, the survival of AML patients remains low, particularly for patients older than age 60 [8,9].

Therefore, there is an absolute need to identify new therapeutic targets and new therapeutic approaches. In this context, an area of growing interest consists in the development of targeted antibody-based immunotherapeutic agents; targets of interest include CD33, CD47, CD70, CD123, FLT3, and CLL-1 for their high expression on the surface of leukemic blasts and leukemic stem cells [10].

## 2. CD123

CD123, the alpha chain of the human interleukin-3 receptor (IL-3R), is a member of the beta common (β_C_) cytokine family, including the GM-CSFR and the IL-5R. These cytokine receptors are characterized by their heterodimeric structure, composed of a specific alpha chain and a common beta chain, which is involved in cell signaling. CD123 expression in normal human hematopoiesis is lineage-specific, in that this receptor is expressed at the level of the majority of CD34^+^ hematopoietic progenitors and its expression is lost during megakaryocytic and erythroid differentiation, while it is maintained in cells differentiating along granulocytic and monocytic lineage [11]; CD123 is expressed only in a part of normal hematopoietic stem cells [12].

IL-3R expression was extensively explored in hematologic malignancies. This receptor is not mutated but frequently overexpressed in several hematological malignancies, including AMLs. Initial studies have shown that CD123 is overexpressed in leukemic CD34^+^/CD38^−^ leukemic stem/progenitor cells compared to the normal counterpart [13]. Testa et al. in 2002 reported the results of a first systematic analysis of CD123 in AML, showing an overexpression in about 45% of cases associated with increased cycling activity of leukemic blasts, increased cellularity (white blood cell count) at diagnosis, and increased responsiveness to cell signaling triggered by IL3 and poor prognosis [14]. These findings were confirmed in subsequent studies [15,16].

AMLs overexpressing CD123 are characterized by some peculiar immunophenotypic features, including low CD34 expression and high CD11b and CD14 expression, and by the frequent occurrence of *FLT3-ITD* mutations [17,18]. Furthermore, a subset of AMLs, characterized by FLT3 overexpression but absent FLT3 mutations, frequently overexpress CD123 [19].

Other studies have confirmed the frequent overexpression of CD123 in *FLT3-ITD*-mutated AMLs and in *NPM1*-mutated AMLs; in particular, Rollins-Raval and coworkers reported a CD123 overexpression in 83% of *FLT3-ITD*-mutated AMLs and in 62% of *NPM1*-mutated AMLs [20]; Brass and coworkers in a large screening on more than 200 AML samples confirmed that the highest CD123 expression was observed in *FLT3-ITD* and *NPM1*-mutated AMLs [21]. Perriello and coworkers analyzed a large cohort of *NPM1*-mutated AML patients and confirmed that CD123 is highly expressed in these leukemias, particularly at the level of the CD34^+^/CD38^−^ cell population [22]. Other studies have confirmed the particularly high expression of CD123 observed in double-mutated *NPM1*-mutated/*FLT3-ITD* AMLs [23].

In *FLT*-ITD-mutant AMLs, CD123 expression was particularly pronounced at the level of the CD34^+^/CD38^−^ leukemic cell population, enriched in LSCs [24,25]. These findings were confirmed in a larger number of FLT3-ITD patients through the isolation of both leukemic progenitors and precursors and showing that both of these cell populations express CD123 [26].

Recent studies carried out in pediatric patients showed that also in some subsets of childhood AML, CD123 expression is particularly pronounced [27]. According to the level of CD123 expression, the leukemic population was subdivided into four CD123 expression-based quartiles: importantly, AMLs with the highest CD123 expression (quartile 4) had a higher prevalence of high-risk *FLT3-ITD* mutations and *KMT2A* rearrangements and lower prevalence of low-risk t(8;21), inv(16) and *CEBPA* mutations [27]. Interestingly, considering *FLT3-ITD*-mutated AMLs, more than 60% display quartile 4 expression [27]. Quartile 4 CD123 expression was associated with poor prognosis (reduced overall survival and event-free survival) compared to quartiles 1–3 expression [27].

A cell population with the CD34^+^/CD38^−^/CD123^+^ phenotype was detectable in about 75% of AMLs [28]. The frequency of these cells in the leukemic blasts is predictive of the clinical outcome [29]. Hermann and coworkers explored the immunophenotypic features of CD34^+^/CD38^−^ cells purified from a large set of AML and CML patients and observed that CD123, as well as CD33, are clearly more expressed on leukemic cells compared to the normal CD34^+^/CD38^−^ counterpart [30].

Vergez and coworkers have extensively investigated the immunophenotypic features of two very large cohorts of AML patients and have identified in these cells six different stages of arrest of leukemic cell differentiation, resembling the features observed for normal hemopoietic progenitor cells: HSC (hemopoietic stem cell, 0.8%), MPP (multilineage progenitor, 21.3%), CMP (common myeloid progenitor, 30.1%), GMP (granulo-monocytic progenitor, 17.4%), MP (monocytic progenitor, 24.2%), and GP (granulocytic progenitor, 6.2%) [31]. The proportion of CD34^+^/CD38^−^/CD123^+^ cells progressively decreases in these AMLs subdivided according to the stage of leukemic arrest [31].

Haubner and coworkers explored the expression levels of surface LSC markers CD123, CD33, CLL1, TIM3, CD244, and CD47 in a large cohort of 356 AML patients at diagnosis, and in 54 of these patients at relapse; CD123, CD244, CLL1, and TIM3 were expressed both at diagnosis and at relapse [32]. This study confirmed also that CD123 expression was higher in *NPM1*-mutated and in *FLT3-ITD* AMLs compared to *NPM1* and *FLT3*-WT AMLs [32].

Houtsma et al. explored the expression of CD123, as well as of other LSC surface markers in CD34^+^ cells of 256 AML samples; a significantly increased CD123 expression compared to normal bone marrow cells was observed in de novo AMLs and in tAMLs but not in sAMLs [33]. The positivity for CD123 and for other LSC markers, such as CD82, CD97, FLT3, IL1RAP, TIM3, and CD25 after two courses of intensive chemotherapy predicted a shorter relapse-free survival [33]. Interestingly, this study showed a significant association between CD123 and CD25 expression in leukemia CD34^+^ cells [33]. Another study showed the occurrence of CD123^+^/CD25^+^ cells in 18% of AML cases, in association with poor outcome (shorter overall survival compared to negative cases) [34].

In conclusion, the expression of CD123 at various stages of the leukemic process from diagnosis to relapse supports CD123 as a suitable therapeutic target for AML.

## 3. General Considerations on CD123 Therapeutic Targeting in Hematologic Malignancies

**Figure 1:**
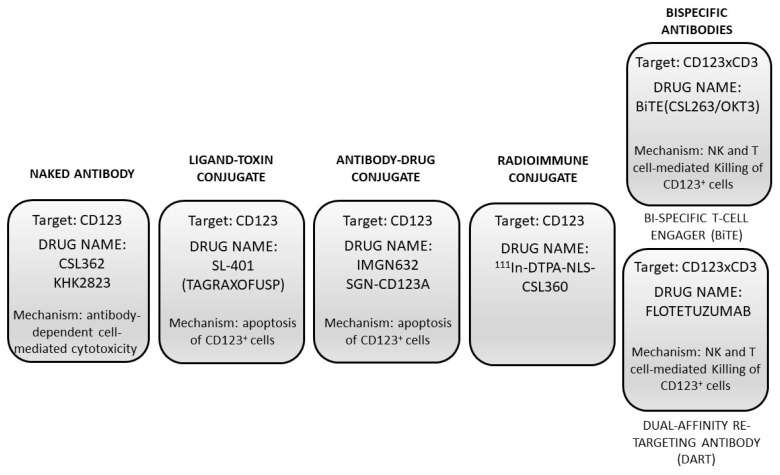
Drugs developed for CD123-targeting therapy in AML and BPDCN.

Preclinical studies have supported the possible therapeutic targeting of CD123 in some hematologic malignancies, such as AMLs and blastic plasmocytoid dendritic neoplasms (BPDCNs) overexpressing this membrane receptor. Since CD123 is just overexpressed and not mutated in hematological malignancies, its therapeutic targeting must involve the use of agents that specifically interact with this receptor, such as its natural ligand IL3, or monoclonal antibodies that either vehiculate cytotoxic agents into CD123^+^ leukemic cells and induce their killing or trigger an immune response, activating the immune system. To this end, numerous agents have been developed, including IL3 conjugated with cytotoxic agents, naked neutralizing anti-CD123 antibodies, antibody–drug conjugates, radioimmune anti-CD123 conjugates, and bispecific antibodies targeting both CD123 and a molecule of the immune system (Figure 1). Bispecific antibodies include a considerable variety of molecular constructs characterized by the property of simultaneous binding of a surface target on leukemic cells (such as CD123) and of molecules like CD3 on T cells, triggering an HLA-independent, immune response against leukemic cells [35]. Basically, three types of bispecific antibodies were generated: (i) Bispecific antibodies (BiTEs) are composed by a single heavy and light chain of the variable region of an antileukemia-associated antigen (CD123) and of CD3, inducing the formation of a cellular complex between T cells, and leukemic cells bridged by a bispecific antibody; in BiTEs, the two antibodies are connected by a linker molecule that contributes to the flexibility of the molecular complex. (ii) Dual-affinity re-targeting molecules (DARTs) have a structure similar to BiTEs but include a disulfide linker to increase the stability of the molecular complex. (iii) Bispecific killer cell engagers (Bikes) and trispecific killer cell engagers (Trikes) are formed by two (Bike) or three (Trike) variable antigen-binding regions and activate natural killer cells though the binding to CD16 (Figure 1) [35].

The development of the technology for chimeric antigen receptor T cell therapy offered a new opportunity of targeting and killing of leukemic cells bearing CD123 on their surface. CARs are different receptors that provide the capacity to T cells to recognize specific tumor antigens and to induce a cytotoxic reaction against tumor cells bearing these antigens and cause their death [36]. CARs are composed by four basic elements: (i) an antigen recognition domain, usually represented by a single-chain variable fragment (scFv) composed of heavy and light chains derived from a monoclonal antibody; (ii) the hinge domain, an extracellular structure bridging the antigen recognition domain and the transmembrane domain; (iii) the transmembrane domain anchoring the CAR to the cell surface membrane; and (iv) the intracellular signaling domain, containing a costimulatory domain and an activation domain [36].

## 4. Ligand–Toxin Conjugates

### SL-401 (Tagraxofusp)

One of the approaches for inducing the selective killing of leukemic cells overexpressing CD123 consisted in the generation of conjugates between the ligand IL-3 and a toxin. In this context, initial studies were based on the generation of a genetically engineered fusion toxin (DT_388_) composed by the first 388 amino acid residues of diphtheria toxin (DT) with a His–Met (H–M) linker, fused to human IL-3, that exerted a pronounced cytotoxic effect against CD123^+^ leukemic blasts [37] and was tolerated in primates up to 100 ug/Kg [38,39]. The level of cytotoxicity exerted on the AML blast directly correlated with the level of CD123 expressed on the surface of these cells [40], and was exerted also at the level of leukemic cells with phenotypic and functional properties of leukemic stem cells [41]. Subsequently, a more potent fusion protein was generated, DT_388_IL-3[K116W], and the resulting compound, SL-401 (tagroxofusp) was introduced in phase I/II clinical studies [42].

Tagraxofusp acts through a two-step process, with a first step involving the IL-3 mediated binding to CD123 and then the internalization, and with a second step involving the cytoplasmic localization of the internalized DT, inducing ADP phosphorylation of the histidine 715 on e2F, with consequent blocking of protein synthesis and finally, cell death.

One of the therapeutic targets of tagraxofusp is represented by blastic plasmocytoid cell neoplasm (BPCDN), a rare hematologic malignancy characterized by the proliferation of leukemic blasts with unique phenotypic features, including also a particularly elevated expression of CD123; at clinical level, this malignancy is characterized by the invasion of some extramedullary compartments implying frequent cutaneous involvement and central nervous system dissemination [43]. This malignancy is associated with poor outcomes and, until recently, the only available treatment consisted of chemotherapy and stem cell transplantation limited to younger patients who achieved a response to chemotherapy [43].

Initial phase I/II studies supported an acceptable safety profile of SL-401, with a maximum tolerated dose of 12.5 ug/Kg/day; a phase II study on 32 BPDCN patients showed 84% of objective responses, with 59% of complete responses [44]. In 2018, the results of a phase II, open label, multicohort study have provided support for the FDA approval of tagraxofusp for the treatment of adult and pediatric BPDCN patients [45] (Table 1). In particular, this study reported the results on 45 BPDCN patients with untreated or relapsed disease treated with 7 or 12 ug/Kg of SL-401 on day 1 or 5 of each 21-day cycle: 29 patients received 12 ug of tagraxofusp as a first-line treatment and 15 as a second-line and third-line treatment [45]. In untreated patients, 72% achieved a complete response and subsequently underwent stem cell transplantation, with a survival rate of 52% at 24 months, while in the treated patients, the response rate was 67% with an overall survival of 8 months [45].

SL-401 was evaluated also in pediatric BPDCN patients. Sun et al. reported the results of three pediatric BPDCN patients treated with SL-401: one of these patients was resistant, while the two others exhibited a significant response to treatment [46]. Recently, Pemmaraju reported the results on eight pediatric BPDCN patients (five patients in first-line treatment and three patients in second- and third-line treatment) treated with tagraxofusp: three out of five first-line patients achieved a complete response and five patients, three in first-line and two in second-line treatment, were bridged to stem cell transplantation [47] (Table 1).

Long-term analysis (median follow-up of 34 months) of the results observed in the clinical study led to the FDA approval of SL-401 for the treatment of BPDCN patients, including also some additional patients (65 treatment-naïve and 19 relapsed/refractory) [11]. For treatment-naïve patients, the ORR was 75%, 57% achieved CR+cCR, and the median duration of response was 24.9 months; 51% of patients achieving a CR were bridged to stem cell transplantation [48]. Capillary leak syndrome occurred in 21% of patients [48]. Furthermore, a sub-analysis of the BPDCN patients enrolled in the phase I/II trial (NCT 02113982) showed that tagraxofusp as a first-line treatment was efficacious in all cohorts of patients, including older patients and patients with significant baseline disease [49].

BPDCN may occur concurrently or with other prior hematologic malignancies (PCHM, 10–20% of patients); therefore, it seemed important to compare the response to tagraxofusp in patients without and in those with PCHM: patients with PCHM (50% of CR) displayed a high rate of response as well as those with no PCHM (58% of CR) [50].

Central nervous system involvement occurs frequently in BPDCN patients, being estimated in the order of 10% at baseline and 30% at first relapse. A recent study showed that tagraxofusp administered concomitantly with intrathecal chemotherapy in patients with BPDCN who either have or are considered at high risk to develop CNS disease is a safe procedure and effective treatment strategy [51].

A recent study retrospectively evaluated 100 BPDCN patients treated either with frontline chemotherapy HCVAD (hyper fractionated cyclophosphamide, vincristine, adriamycin, and dexamethasone, 35 patients) SL-401 (37 patients) or other regimens (28 patients), showing the following results: overall survival 28.3 vs. 13.7 vs. 22.8 months, respectively; complete remission rate 80% vs. 50% vs. 43%; rate of stem cell transplantation 51% vs. 49% vs. 38% [52]. These observations supported a continued value of HCVAD-based chemotherapy in BPDCN, even in the actual targeted-therapy era [52].

Lane et al. reported the preliminary results of a phase Ib study exploring the safety and efficacy of combining tagraxofusp with azacitidine, or azacitidine and venetoclax, in CD123-positive AML, MDS, or BPDCN patients; in this report, three relapsing/refractory BPDCN patients were included and were treated with the triplet tagraxofusp, azacitidine, and venetoclax: two of these patients responded to this treatment, achieving a CR or CRi response and were bridged to allo-stem cell transplantation [53] (Table 1). The addition of the BCL2 inhibitor venetoclax to azacitidine and tagraxofusp is fully justified by several recent studies showing both at the preclinical and clinical level the sensitivity of BPDCN to venetoclax used either as monotherapy or in combination with azacitidine [54,55,56].

Another set of clinical studies explored the possible use of tagraxofusp in the therapy of AMLs. The results of a phase I clinical study carried out in 45 AML patients receiving a single infusion of tagraxofusp showed a low rate of responding patients, limited to one CR and two PRs [57] (Table 1). More recently, Lane and coworkers reported the results of a phase Ib study evaluating the safety and the efficacy of tagraxofusp administered in combination with azacytidine or azacitidine and venetoclax. Fourteen AML patients were treated with tagraxofusp plus azacitidine: in five AML patients in first-line treatment, one CR was observed, and in nine relapsing/refractory AML patients, no objective responses were observed [53]. Twelve AML patients were treated with tagraxofusp, azacytidine, and venetoclax: in nine patients in first-line treatment, five complete responses and three complete responses with incomplete hematological recovery were observed; in nine relapsing/refractory AML patients, no objective responses were observed [53] (Table 1). Importantly, 50% of the responding patients were bridged to stem cell transplantation; among the responding patients, two had *TP53* mutations and adverse karyotype, and three had secondary AMLs [53].

An ongoing clinical trial is evaluating the response to tagraxofusp plus azacitidine of secondary AML patients who have failed to respond to hypomethylating agents [58]. Another ongoing clinical trial is evaluating the capacity of tagraxofusp in combination with azacytidine to eradicate minimal residual disease prior to allogeneic stem cell transplantation [59].

A significant proportion of leukemic patients are resistant to tagraxofusp and other patients develop resistance after an initial response; therefore, the presence or the development of resistance mechanisms represents a major limitation to CD123 targeting through tagraxofusp. Stephanski and coworkers have shown the existence of a resistance mechanism not related to CD123 expression. In fact, in both sensitive and resistant leukemic cells, SL-401 treatment does not alter CD123 expression. Furthermore, they observed in resistant leukemic cells a downregulation of the expression of DPH1 (diphthamide biosynthesis 1), the enzyme responsible for the conversion of histidine 715 on eEF2 to diphthamide, the direct target of ADP ribosylation diphtheria toxin [60,61]. The exploration of leukemic blasts of patients treated with SL-401 and resistant to this drug showed decreased DHP1 expression; furthermore, downmodulation of DHP1 expression in leukemic cells decreased their sensitivity to SL-401 [62,63]. Interestingly, azacitidine, a DNA methyltransferase inhibitor used in the treatment of AMLs, induced a reversion of DHP1 expression and restored the sensitivity to SL-401 [60,61]. These studies suggest that the combination of tagraxofusp with azacitidine could represent a rational drug association in the treatment of BPDCN. Preliminary studies based on few BPDCN patients support this hypothesis. Thus, a case report showed the successful treatment with tagraxofusp and azacitidine of an older patient with relapsed BPDCN after allogeneic stem cell transplantation [62].

A recent case report described a new mechanism of development of tagraxofusp resistance related to a marked downmodulation of CD123 expression in BPDCN relapsing leukemic cells [63].

## 5. Naked Antibodies

### CSL362

Unmodified monoclonal antibodies were explored at therapeutic level for their capacity to inhibit the binding of IL3 to its receptor and to activate innate immune mechanisms mediating the clearing of cells expressing CD123. An example of these antibodies is represented by the neutralizing anti-CD123 7G3 monoclonal antibody, first humanized and affinity matured, and then engineered at the level of the Fc-domain to potentiate its cytotoxicity-inducing capacity against AML cells; the antibody thus modified was called CSL362 and was explored first in preclinical studies and then in clinical trials in AML patients [64]. Preclinical studies have shown the capacity of CSL362 to target CD123^+^ AMLs and to induce ADCC-dependent lysis of AML blasts [65]. CSL362 was evaluated in a phase I clinical trial in 40 AML patients with refractory/relapsing disease and only two of these patients displayed a clinical response [66]. A second phase I study was performed in a cohort of AML patients achieving a first or second remission but who were not candidates for stem cell transplantation: 11 of these patients displayed minimal residual disease that, in four cases, was converted to negativity after treatment with CSL362 [67].

However, two more recent studies failed to show a significant benefit in AML patients treated with CSL362 (investigated with the commercial name of talacotuzumab) [68,69].

Following these negative results, the program of clinical development of talacotuzumab was discontinued by Johnson and Johnson Company, New Brunswich, New Jersey, USA.

## 6. Antibody–Drug Conjugates

### 6.1. IMGN632

Miller et al. developed a procedure for the generation of antibody–drug conjugates (ADCs) with high therapeutic indices and favorable toxicities [70]. This strategy was based on a new DNA cross-linking agent (DNA-cross-linking pyrrolobenzodiazepine compounds as the payload) [70]. A further improvement of this technology consisted in changing the mechanism of action from a cross-linker to a DNA alkylator: this change resulted in the development of ADCs containing the DNA alkylator with similar in vitro potency, but improved bystander killing and in vivo efficacy, compared with those of the cross-linker [71]. Using this technological approach, a CD123 antibody 4723A was linked to DNA mono-alkylating payload of the indolinobenzodiazepine pseudodimer (IGN) class of cytotoxic compounds [72]. The activity of IMGN632 was compared with X-ADC, the ADC compound developed utilizing the G723A antibody linked to a DNA cross-linking IGN payload. Both of these compounds, IMGN632 and X-ADC, exhibited the same potency against AML cell lines and primary AML blasts, but X-ADC exposure was >40-fold more cytotoxic to the normal myeloid progenitors than IMGN632 [72]. Importantly, IMGN632 displayed a cytotoxic effect on AML samples at doses not exerting negative effects on normal myeloid progenitors [72].

Initial studies have reported the evaluation of IMGN632 as a monotherapy in AML patients with relapsing/refractory disease [73]. In the evaluable AML population composed by 66 patients, 55% of them displayed a reduction of leukemic blasts, and 20% an objective response; most responders failed prior intensive therapies [73] (Table 1).

Preclinical studies have shown a consistent antileukemic synergism of the combination of IMGN632 and venetoclax, a BCL-2 inhibitor approved for elderly AML patients in combination with azacitidine [74]. The synergistic interaction between IMGN632 and venetoclax was shown both in leukemic cell lines and in AML PDX (patient-derived xenograft) [74]. These observations have supported clinical studies involving the administration of IMGN632 in combination with venetoclax and azacitidine. A phase Ib/II study was designed to evaluate the safety and the efficacy of a triplet regimen based on IMGN632, azacitidine (AZA), and venetoclax (VEN): the triplet escalation phase of the study involved five cohorts of patients, four cohorts dosed IMGN632 on day seven of each cycle and one cohort dosed IMGN632 on day one of each cycle [75]. A recent report on this study at the ASH Meeting, December 2022, reported the preliminary safety data on 71 AML patients with relapsed/refractory disease (30% with secondary AML, 32% with primary refractory disease; 44% received prior VEN treatment and 22% had prior allogeneic bone marrow transplant) and the efficacy data on 61 patients: concerning safety, the treatment was well tolerated with 30% of patients displaying febrile neutropenia and 21% infusion-related reactions; concerning efficacy, 51% of patients displayed an objective response, with 31% complete responses [76] (Table 1). Interestingly, VEN-naïve patients exhibited an ORR and CCR of 62% and 47%, compared with 37% and 11%, respectively, in patients with prior VEN treatment [76].

Other clinical studies have explored the efficacy of IMGN632 in patients with BPDCN. Thus, Naver et al. explored in a preliminary study the efficacy of IMN632 in seven relapsing/refractory BPDCN patients and reported that three of these patients achieved objective responses, two patients had stable disease, and two patients had clinical progression; interestingly, the three patients exhibiting an objective response received prior treatment with SL-401 [73] (Table 1). The CADENZA clinical trial (NCT 03386513) is enrolling two expansion cohorts for adult patients with CD123-positive BPDCN: one cohort is enrolling patients with frontline/untreated BPDCN disease; the other cohort is enrolling patients with relapsed/refractory BPDCN which may have had up to three lines of prior therapy, including CD123 targeting and stem cell transplantation [77]. The patients were treated at the IMGN632 dose of 0.045 mg/kg once every three weeks. An update provided by Immunogen Company on September 2022 on the first patients treated in the context of the CADENZA trial reported that: (i) four patients with de novo BPDCN treated with IMGN632 showed a CR or a clinical CR; (ii) of the six patients with BPDCN who had prior or concomitant hematologic malignancy, four experienced a CR, clinical CR, or CR with partial hematological recovery [78]. The final results of this study are expected for the end of 2024.

### 6.2. Other ADCs

Another study reported the development of SNG -CD123A, an ADC CD123 antibody obtained using the pyrrolobenzodiazepine dime (PBD) linker and a humanized CD123 antibody with cysteines for site-specific conjugation [79]. SGN-CD123A induced apoptosis of CD123-expressing leukemic cells and resulted to be active against myeloid leukemic cell lines and primary AML blasts and in patient-derived xenograft models [79].

Han and coworkers reported the development of optimized ADC based on a novel DNA-damaging payload, cyclopropa[c]pyrrolo[3,3-e]indole-4 one dimer (CPI dimer), that is bound to an engineered Y296Q residue in the antibody heavy chain via transglutaminase mediated conjugation, enabling an ADC preparation consisting of a homogeneous load of drug to antibody ratio of two [80]. Using this approach, it was shown that this platform allows the production of ADC with a higher therapeutic index [80]. Furthermore, the head-to-head comparison of two AML surface proteins, CD123 and CD33, showed that CD123-ADCs display an efficacity comparable to that of CD33-ADCs, but a better safety profile in nonhuman primates [80].

## 7. Bispecific Antibodies

### 7.1. Flotetuzumab

The bispecific antibody binds to a tumor-specific antigen using one epitope and to immune cell receptors such as CD3, CD16, CD64, and CD89 with the other epitope, with the objective of stimulating cell immune responses against tumor cells. Initial studies have reported the generation of bispecific antibodies with specificities for the extracellular domain of CD3 and for the N-extracellular domain of CD123. The antibodies induced both in vitro and in vivo activation of T lymphocytes and killing of CD123^+^ leukemic blasts [81,82]. In 2015, MacroGenics (Rockville, MD, USA) reported the development of MGD 066, a CD3xCD123 DART (dual-affinity re-targeting) protein composed of anti-human CD123Fv and humanized mouse anti-human CD3; this molecule exerted a potent antileukemic activity both in vitro and in vivo [83,84]. The preclinical studies carried out using MDG-006 supported the development of a clinical program of evaluation of this molecule, introduced with the commercial name of flotetuzumab. A phase I study enrolled 30 AML patients with refractory/relapsed disease, providing evidence of antileukemic activity in 67% of patients, with 19% of complete responses (31% among patients with refractory disease, but 0% in patients with relapsing disease) [85] (Table 1). Analysis of the immune activation status in these patients suggested that an immune-enriched gene signature could correlate with response to flotetuzumab [86,87].

In 2021, Uy et al. reported the results of a phase I/II study on 88 AML patients with refractory/relapsed disease treated with flotetuzumab as salvage therapy: 42 patients pertains to a dose-finding segment and 46 to a group treated with the recommended dose of flotetuzumab (500 mg/kg/day) [88]. Of these patients, 26.7% displayed a complete remission/complete remission with incomplete hematological recovery; the overall survival in patients who achieved a CR was 10.2 months [88].

Vadakekolathu and coworkers, through the analysis of three large cohorts of AML patients, identified immune-infiltrated and immune-depleted classes of AMLs; they also identified interferon-γ-related mRNA profiles that were predictive of chemoresistance and of response to flotetuzumab immunotherapy [89].

Interestingly, the study of *TP53*-mutated AMLs, a subgroup associated with a very poor outcome, showed that these leukemias are characterized by a higher expression of IFN-γ, FOXP3, immune checkpoints, and markers of immune senescence; seven out of 15 patients (47%) with refractory/relapsed AML and *TP53* abnormalities achieved a complete response following immunotherapy with flotetuzumab [90]. *TP53*-mutated AML patient responders to flotetuzumab therapy had significantly higher tumor inflammation signature, FOXP3, CD8, inflammatory chemokine, and PD1 gene expression scores at baseline compared with non-responders [90].

Recent preclinical and clinical studies have explored the use of flotetuzumab in pediatric AML. At preclinical level, the efficacy of fotetuzumab was shown in combination with cytarabine in PDX models of pediatric AML [91]. A recent phase I trial reported the safety profile and preliminary activity of flotetuzumab in pediatric patients with relapsed/refractory AML: flotetuzumab appeared to be tolerable in these patients at the recommended dose of 500 ng/kg/day and induced 20% of responses, including CR+PR [92].

A recent study based on the analysis of primary AML blasts of AML patients relapsed after hematopoietic stem cell transplantation incubated in vitro with flotetuzumab or of refractory/relapsed AML patients treated in vivo with flotetuzumab showed an increase in major histocompatibility class II (MHC-II) expression induced by IFN-γ production [93]. These observations suggest that flotetuzumab may induce the killing of refractory/relapsed AML cells through MHC-dependent mechanisms mediated through local increase of IFN-γ [93].

### 7.2. MGD024

Recently, MacroGenics reported the development of a new CD3-engaging bispecific molecule targeting CD123, MGD024 [94]. MGD024 is an Fc-bearing CD123xCD3 DART molecule designed for prolonged circulating half-life and intermittent delivery; furthermore, MGD024 was engineered with a CD3-binding arm exhibiting reduced affinity to decrease the tendency to induce cytokine release compared to flotetuzumab [94]. At structural level, MGD024 was identical to flotetuzumab, sharing identical CD123 and CD3 Tv arms, with the exception for the introduction of a mutation in the anti-CD3 arm of MGD0124 decreasing its affinity for the CD3-epsilon chain; however, at variance with flotetuzumab, MGD024 contains an Ala-Ala mutated human IgG1 Fc, extending its circulating half-life [94]. In preclinical models, MGD024 demonstrated reduced in vitro and in vivo potency compared to flotetuzumab and requires the administration of higher doses compared to flotetuzumab; furthermore, reduced cytokine release was observed with MGD024 compared to flotetuzumab [93]. Studies in animal models of AML have supported the possible co-administration of MGD024 with cytarabine or venetoclax or azacitidine, showing complete or near-complete elimination of tumor cells using the combination of MGD024 with cytarabine or with venetoclax [94]. These observations have supported the clinical exploration of MGD024 in refractory/relapsed AML patients. Thus, a classical phase I dose-escalation study was proposed to evaluate the safety of MGD024 in some refractory/relapsed hematologic malignancies, including AML and BPDCN [95].

### 7.3. Other Bispecific Antibodies

Other bifunctional CD3-CD123 monoclonal antibodies have been developed and are under evaluation in AML clinical trials. XmAb14045 (known by the commercial name vibecotamab) is a bispecific antibody targeting both CD123 and CD3 that stimulates targeted T cell-mediated killing of CD123-expressing cells; at variance with other bifunctional constructs (DART or BiTE), XmAb14045 is a full-length immunoglobulin molecule designed to be dosed intermittently [96]. A phase I clinical study enrolled 104 relapsed/refractory AML patients treated with vibecomatab dosages from 0.003 to 12 ug/Kg; in the group of patients treated at higher dose levels (0.75 ug/Kg), a 14% overall response rate was observed, including also five CRs [97] (Table 1). Biomarker analysis suggested that responding patients harbored a lower burden of disease and specific T cell subtypes [97] (Table 1).

APV0436 is a humanized bispecific antibody that targets both CD123 and CD3. It is composed of two binding domains linked to a human IgG1 Fc domain. The CD123 binding domain is a fully human scFv directed against human CD123; the CD3 binding domain is a humanized scFv that binds human CD3; the Fc region has been engineered to minimize complement fixation and interaction with Fcγ receptors [98,99]. Preclinical studies in primates have shown the in vivo efficacy and the safety of APV0436, thus supporting a program of clinical development. A phase Ib clinical study supported the safety and defined the optimal dose for subsequent studies (0.2 ug/Kg) of APV0436 and provided preliminary data on its efficacy in a population of relapsed/refractory AML and MDS patients [100]. A recent report showed the preliminary results of the expansion phase of this phase Ib study; the expansion phase of this trial was complex and involved five cohorts of patients: cohorts three and five APV0436 in monotherapy, while cohorts one, two, and four involve combination therapy of APV0436 with chemotherapy, venetoclax, and azacitidine, respectively [101]. Preliminary results are available only for cohorts one, two, and three with a rate of response of 33%, 40%, and 20%, respectively [101].

A first-in-human study evaluated the safety and the clinical efficacy of JNJ-63709178, a CD123/CD3-targeting antibody, in a group of relapsed/refractory AML patients; either intravenous or subcutaneous administration of the antibody was evaluated [102]. Both intravenous and subcutaneous dosing of JNJ-63709178 were associated with suboptimal drug exposure, unfavorable safety profiles, and limited clinical efficacy [102].

A recent study reported the development of a bispecific antibody, IGM-2537, a novel IgM antibody-based CD123 × CD3 bispecific T cell engager, designed to potentiate the antitumor activity and to lower cytokine release; this molecule has 10 binding sites for CD123, and a single binding site for CD3 through single chain Fv domain (scFv) fused to a joining chain, thus allowing high-affinity and high-avidity binding to CD123-positive leukemic cells and engagement of CD3 T cells inducing T cell-induced cytotoxicity with reduced cytokine release [103]. In vitro and in vivo assays showed a potent antileukemia activity with minimal cytokine induction [103]. These observations support a program of clinical development for this molecule.

A trispecific molecule is represented by SAR443579, a trifunctional natural killer cell engager (NKCE) targeting CD123 on leukemic cells and CD16a and NKp46 on NK cells [104]. Preclinical studies have shown that SAR443579 induced potent antileukemic activity against AML blasts, promoted NK cell activation, and induced cytokine release in the presence of AML cells [104]. A phase I study was proposed to evaluate the safety and the efficacy of SAR443579 in relapsed/refractory AML and B-ALL patients [104].

## 8. CD123 Chimeric Antigen Receptor T Cell Therapy

Antitumor adoptive cellular therapies represent an important tool in the treatment of tumors. In this context, studies carried out in the last decade have evaluated the safety and potential therapeutic impact of adoptive cellular therapies based on the use of genetically engineered cells. Two different types of therapies have been developed using genetically modified T cells: (i) T cell receptor-engineered cells enabled to recognize specific membranes in a HLA-constricted context; (ii) CART transduced T cells that interact with specific membrane antigens in an HLA unrestricted and antibody-specific manner [105].

**Figure 2:**
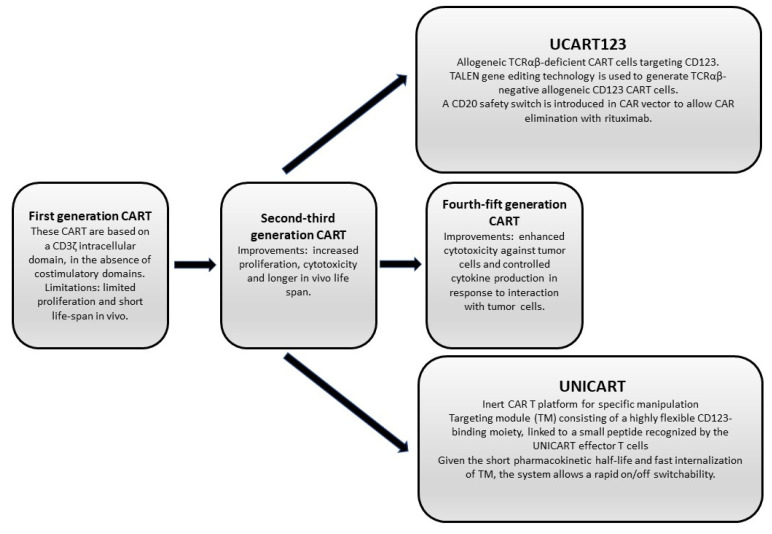
Different types of CD123 CART cells developed for CD123-targeting therapy in AML and BPDCN.

The procedure of generation of CAR-T cells evolved over time with five different CAR-T generations, from the first procedures in late 1990 to the most recent developments [105] (Figure 2). The first generation of CAR-T was based on CD3ζ intracellular signalling domain, in the absence of costimulatory domains; the second generation of CAR-T cells contained in the intracellular domain a costimulatory domain, such as CD28; the third generation was based on the presence of multiple costimulatory domains; the fourth generation involved the production of T cells redirected for general universal cytokine-mediated killing(TROCKs), a property obtained through IL-12 production, either constitutive or after CAR-T activation; the fifth generation included also a STAT3 binding site required for the generation of three activation signals acting on the cell signalling, costimulatory and cytokine signalling domains [105]. The last generations of CAR-T cells showed a superior in vivo persistence and antitumor effects in models of leukemia or of solid tumors as compared to initial CAR-T cell generations and are expected to demonstrate superior antitumor effects with reduced toxicity in the clinic [106].

Mardiros et al. reported the development of second-generation CD123 CAR-T cells using a vector containing a single-chain variable fragment, a IgG4 linker, a CD28 co-stimulatory domain, and a CD3 zeta domain used to engineer either autologous or donor-derived T lymphocytes; these cells exhibited potent effector activity against primary AML cells and exerted only a limited cytotoxicity in vitro against hematopoietic progenitor cells [107]. Using this methodology, Mustang Bio Inc generated a clinical drug called MB02 and evaluated it in the context of a phase I clinical trial; nine patients (seven refractory/relapsed AML patients previously receiving allogeneic stem cell transplantation and two BPDCN patients were treated with MB-02: 2 AML patients were treated at dose zero (50 × 10^6^ CAR-T) and one of the two achieved a morphologic leukemic-free state; five AML patients were treated at dose one level (200 × 10^6^), with one patient achieving complete remission with incomplete marrow recovery, another patient showing a morphologic leukemic-free state and three patients displaying stable disease; two BPDCN patients were treated at dose zero, with one patient achieving a complete response [108].

In 2017, Cartellieri and coworkers reported the development of a novel modular universal CAR platform technology called UniCAR that allows a reduction of the risk of on-target side effects of CAR-T by inducing a rapid and reversible control of CAR-T cell reactivity [109]. Two components are required in the UniCAR system: (i) a CAR for an inert manipulation of T cells; and (ii) specific targeting modules for redirecting UniCAR-T cells in a personized time-independent and target-dependent manner [109]. The rapidly switchable universal CAR-T platform was adapted to target CD123 and the resulting 123-UniCART: these 123-UniCAR exerted in vitro and in vivo in patient-derived xenograft models a marked antileukemic activity; furthermore, using a hematotoxicity mouse model, it was shown that CD123UniCAR exerted reversible toxicity toward hematopoietic cells compared to CD123CAR-T [110,111]. These UniCAR-T CD123 cells were evaluated in a phase I clinical trial (NCT 04230265) in relapsed/refractory AML patients with the aim of evaluating the safety profile and for obtaining preliminary data on clinical efficacy. In 2021, the results on the first three treated patients were reported; the treatment schedule involved UniCAR-T cell administration on day 1 and intravenous infusion of soluble TM123 from days 0 to 24 [112]. All three patients displayed a clinical response, with two patients achieving complete remission with incomplete hematologic recovery and one patient showing a partial response [112]. The results of the first 14 AML patients treated with UniCAR2-T-CD123 were recently reported at the 2022 ASH Meeting showing that: (i) the treatment was generally well tolerated with a limited number of treatment-related adverse events; (ii) 10 patients showed a decrease in blast cell counts, including two CRi, one patient with MRD positive CR converted to level of negativity, and four PR [113] (Table 1). These results supported further clinical investigation, with an implementation of expansion cohort at the most appropriate dosage [113].

Qin et al. reported a new methodology to improve the quality of targeting domains included into the structure of CAR-T: using this approach, they identified and characterized domains specific for CD123 and incorporated these domains into CAR-T exhibiting potent T cell activation and cytolysis of CD123-expressing leukemic cells, inducing complete durable remission in two AML xenograft models [114].

Epigenetic modulators used for AML treatment modulate the activity of CD123 Car-T cells. Thus, You et al. showed in preclinical AML models that decitabine enhances the antileukemia efficacy of CD123 CAR-T cells in vitro and in vivo [115].

The use of allogeneic T cells for the generation of CD123-CAR-T cells is obviously associated with the risk of generating a graft vs. host disease. To decrease this risk, TALEN gene-editing technology was used to produce a TCRαβ-negative allogeneic CD123 CAR (UCART 123); these CAR-T cells preferentially eliminate AML over normal cells with minimal toxicity to normal hematopoietic stem/progenitor cells [116]. Furthermore, as a safety feature, these cells were engineered to express RQR8 to allow their elimination with rituximab [116].

UCAR123 v1.2 was evaluated in the context of the AMELI-01 phase I clinical trial involving the enrolment of relapsed/refractory AML patients; UCART123 v1.2 was administered by escalating dose after lymphodepletion with either fludarabine and cyclophosphamide (FC), or FC plus alemtuzumab in patients with relapsed/refractory AML. Incomplete lymphodepletion was achieved with FC. In the eight patients lymphodepleted with FC 1, stable disease and one morphological remission was achieved; in the eight patients lymphodepleted with FCA I, stable disease and one complete remission with MRD-negativity (persisting after 8 months of follow-up) were observed [117].

A recent study reported the first results of a Phase I clinical trial investigating the safety and efficacy of autologous third generation CD123 CAR-T cells generated with a vector containing a CD123-CAR with a CD28 signaling domain and a CD20 safety switch in a population of pediatric AML patients with relapsed/refractory disease [118] (Table 1). The treatment schedule involved four treatment dosages: DL1 at 3 × 10^5^ cells/Kg, DL2 at 1 × 10^6^ cells/Kg, DL3 at 3 × 10^6^ cells/Kg, and DL4 at 1 × 10^7^ cells/Kg [118]. In two patients infused on DL1, no responses were observed; in three patients infused on DL2, no response was observed in one patient, reduction in blast percentage in one patient, and complete remission in one patient were observed [118]. CD123 CAR-T cell expansion in patients on DL2 but not in those on DL1 was observed [118].

HLA-independent T cells have emerged as a promising candidate for their peculiar immunological properties, related to their cytotoxic activity, release of immunostimulating cytokines, and recruitment of other immune cells to the tumor site. Thus, Martinez et al. reported the development of CD123 CAR-T delta one T (DOT) cells using γδ T lymphocytes transduced with a 4-1BB-based CAR DOT directed against the IL-3Rα [119]. CD123 CAR-DOT cells displayed a pronounced cytotoxic activity against CD123-positive leukemic cell lines and primary blast cells, superior to control CD123 CAR-T cells [119]. Alternatively, Caruso et al. reported the generation of allogeneic NK cells engineered to express a second-generation CAR targeting CD123: these CD123 CAR-NK cells displayed a strong cytotoxicity in vitro against primary AML blasts and in vivo in immunodeficient mice AML models [120]. Importantly, CD123 CAR-NK cells are clearly less cytotoxic than CD123 CAR-T cells against normal bone marrow cells, as evaluated in immunodeficient mice grafted with human hematopoietic cells [120].

Finally, Boucher et al. have recently reported the generation of CD123/CD33 bispecific CAR-T cells that seem to have a considerable therapeutic potential and will be evaluated in clinical trials in relapsed/refractory AML patients [121].

Several attempts have been pursued in the development of fourth-fifth generation CAR-T cells. Transgenic expression of IL15, a γ-cytokine, in CAR-T cells retains these cells in a less differentiated state and improves their expansion and in vivo survival in a preclinical xenograft model [122]. Particularly, CD123 CAR-T cells engineered to secrete IL15 displayed higher anti-AML activity, remained in a less differentiated state, and showed a significant survival advantage in AML xenograft and in autologous patient-derived xenograft models [123]. It is of interest to note that, despite the improved anti-AML activity of CD123-CAR-IL15-T cells, leukemia eventually progressed in PDX-AML models; this late therapeutic failure seemed to be due to a decreased CD123 expression post CD123 CAR-T cell therapy [123]. This important observation implies two considerations derived from current studies on CAR-T cells: (i) optimal antigen density on tumor cells is a fundamental requirement for CAR-T cell activity and strategies for enabling CAR recognition of low antigen cells are required [123]; (ii) preclinical studies have shown that strategies to target multiple antigens expressed in AML cells are needed to achieve optimal therapeutic responses [124,125]. To generate CAR-T cells with multiple antigenic reactivity, a strategy was proposed based on the transduction of T cells with a synthetic agonistic receptor (SAR) composed of an inert extracellular domain (EGFRvIII) acting as an antigen receptor fused to intracellular T cell-activating domains that can be specifically activated by an engineered bispecific antibody [126,127]. This procedure allowed the generation of SAR-CAR-T specific for CD123 and CD33 [127]. Furthermore, AML cells have a direct immunosuppressive effect that limits in many patients the success of CAR-T cell-mediated therapy [128]. T cell derangements (immune exhaustion and senescence) are common in AML and affect the response of these patients to chemotherapy, molecularly targeted therapies, and immunotherapies [129].

## 9. Conclusions

The first-in-class CD123-targeting therapy, tagraxofusp, has shown its efficiency and was approved for treatment of BPDCN. The efficacy of this therapy is well evident; however, the results obtained in terms of overall survival are still not optimal and comparable to those that can be achieved with intensive chemotherapy. Therefore, other CD123-targeting strategies are in development, including bispecific antibodies and combination therapies including tagraxofusp; adoptive T cell therapy using CAR-modified T cells targeting CD123 are under evaluation in BPDCN patients.

The preclinical and clinical studies have shown that the targeting of CD123 is a rational approach for the treatment of some hematologic malignancies, including AML. The chances to obtain complete eradication of the leukemic process using only CD123 targeting as a therapeutic approach are mainly dependent on the expression of IL-Rα at significantly elevated levels on the surface of all leukemic cells that can maintain the leukemic process. The results obtained at clinical level using the various agents targeting CD123 as monotherapy have shown their incapacity to completely eradicate the leukemic process in the patients responding to this treatment; this failure may be due to various mechanisms: (i) a part of leukemic cells does not express CD123; (ii) a part of leukemic cells express CD123 at low/very low levels that cannot be efficiently targeted; and (iii) leukemic cells present at the level of body compartments escape CD123 targeting because they are not efficiently reached by CD123-targeting agents.

Particularly interesting are the results observed with the bispecific antibody flotetuzumab in *TP53*-mutated AML patients [89,90]. In fact, tumor inflammation signature, IFN-γ pathway, chemokines, and lymphoid signature scores were higher in *TP53*-mutated AMLs than in complex karyotype AMLs with WT-*TP53*; furthermore, expression of immunosuppressive genes, such as *FOXP3*, *IFNG*, *CD8A*, *LG3*, *and GZMB* and of immune checkpoints (PD-L1 and TIGIT), is high in *TP53*-mutated AMLs [89,90]. These observations have suggested that *TP53*-mutant AMLs have an immunosuppressed tumor microenvironment, a condition that makes these leukemias more sensitive to immunotherapy with flotetuzumab [89,90]. These observations must be confirmed in a larger cohort of *TP53*-mutated AMLs and the therapeutic effects obtained with flotetuzumab need to be consolidated through an association of flotetuzumab with other antileukemia agents active against *TP53*-mutant AMLs.

Future studies of CD123 targeting in AML patients have to carefully consider all these elements and should be based on: (i) the evaluation of combination therapies based on the use of a CD123-targeting drug with antileukemic drugs such as venetoclax or chemotherapy both in first-line and second-line treatment; (ii) the evaluation of CD123-targeting therapies in selected subpopulations of AML patients that are expected to be more sensitive to these treatments; (iii) the evaluation of CD123-targeting therapies in an attempt to eradicate the minimal residual disease; and (iv) the development of strategies to reduce the toxicity of CD123-targeting therapies.

## Figures and Tables

**Table 1:** Clinical trials in BPDCN and AML patients involving CD123 targeting. Abbreviations: AZA: azacitidine; VEN: venetoclax; CR: complete remission; PR: partial remission; R/R: relapsed/refractory; FL: front line.

Target	Drugs	Phase	Disease	Outcome	Adverse Events
CD123	Tagraxofusp	III	BPDCN 32 untreated 13 prev. treated	CR+CRi 54%; in untreated patients 72%; 52% OS at 24 months	Capillary Leak Syndrome 21%
CD123	Tagraxofusp	I	40 R/R AML 5 R/R MDS	AMLs: CR 2.5%; PR 2.5% MDS: PR 2.5%	Capillary Leak Syndrome 31%
CD123 BCL2	TAG+AZA TAG+AZA+VEN	I	14 AML (FL) 12 R/R AML 3 R/R BPDCN 4 MDS	TAG-AZA: AML (FL) CRi 20%; R/R AML CR 0% TAG-AZA-VEN: AML (FL) CR+CRi 89%; R/R AML CR 0%; R/R BPDCN CR+CRi 66%	Capillary Leak Syndrome 33%
CD123	IMGN632 (Pivekimab Sunirine)	I	67 R/R AML	CR+CRi 20%	Cytokine Release Syndrome 16%
CD123 BCL2	IMGN632 AZA VEN	Ib/II	35 R/R AML	CR+CRi 31%	Cytokine Release Syndrome 37%
CD123	IMGN632	I	23 R/R BPDCN	CR+CRi 22% PR 8%	Cytokine Release Syndrome 22%
CD123 CD3	APV0436 (BiTE)	I	22 R/R AML, either pAML or sAML	CR+CRi 32%	Cytokine Release Syndrome 18%
CD123 CD3	XmAb 14045 (Vibecotamab) (BiTE)	I	104 R/R AML	CR+CRi 14% (evaluated in 51 patients at optimal dose)	Cytokine Release syndrome 59%
CD123 CD3	Flotetuzumab (DART)	I/II	88 R/R AML	CR+CRi 30% (evaluated in 46 patients at optimal dose)	Cytokine Release Syndrome 13%
CD123 CD3	Flotetuzumab (DART)	I	17 R/R pediatric AML	CR+CRi 12% PR 6%	Cytokine Release Syndrome 9%
CD123	UniCAR-T	I	14 R/R AML	Blast cell count reduction (10 patients), CRi (2 patients), CR with MRD negativity (1 patient)	Cytokine Release Syndrome (12/14)
CD123	Anti-CD123 allogeneic CAR-T	I	16 R/R AML	SD (2/16), blast cell count reduction (1 patients), CR with MRD negativity (1 patient)	Cytokine Release Syndrome (15/16)
CD123	CAR-T	I	12 R/R pediatric AML	Blast cell count reduction (1 patient), CR (1 patient)	No grade 2 cytokine release syndrome

## Data Availability

No new data were created or analyzed in this study. Data sharing in not applicable to this article.
